# Nitrite inhalant use among young gay and bisexual men in Vancouver during a period of increasing HIV incidence

**DOI:** 10.1186/1471-2458-7-35

**Published:** 2007-03-15

**Authors:** Thomas M Lampinen, Kelly Mattheis, Keith Chan, Robert S Hogg

**Affiliations:** 1 Department of Health Care and Epidemiology, University of British Columbia, Vancouver, Canada; 2 BC Centre for Excellence in HIV/AIDS, Vancouver, Canada; 3 Department of Family Practice, Faculty of Medicine, University of British Columbia, Vancouver, Canada; 4 Faculty of Health Sciences, Simon Fraser University, Burnaby, Canada

## Abstract

**Background:**

Nitrite inhalants ("poppers") are peripheral vasodilators which, since the beginning of the epidemic, have been known to increase risk for acquiring HIV infection among men who have sex with men (MSM). However, few studies in recent years have characterized use. From 1999 to 2004, new HIV diagnoses among MSM in British Columbia increased 78%, prompting us to examine the prevalence and correlates of this modifiable HIV risk factor.

**Methods:**

Self-administered questionnaires were completed between October 2002 and May 2004 as part of an open cohort study of HIV-seronegative young MSM. We measured nitrite inhalant use during the previous year and use during sexual encounters with casual partners specifically. Correlates of use were identified using odds ratios.

**Results:**

Among 354 MSM surveyed, 31.6% reported any use during the previous year. Nitrite inhalant use during sexual encounters was reported by 22.9% of men and was strongly associated with having casual partners, with greater numbers of casual partners (including those with positive or unknown serostatus) and with anal intercourse with casual partners. Nitrite inhalant use was not associated with non-use of condoms with casual sexual partners per se.

**Conclusion:**

Contemporary use of nitrite inhalants amongst young MSM is common and a strong indicator of anal intercourse with casual sexual partners. Since use appears to increase the probability of infection following exposure to HIV, efforts to reduce the use of nitrite inhalants among MSM should be a very high priority among HIV prevention strategies.

## Background

Nitrite inhalants ("poppers") are peripheral vasodilators used by men who have sex with men (MSM) to facilitate and enhance sexual intercourse [1]. Throughout the AIDS epidemic, studies have associated use of nitrite inhalants among MSM with prevalent HIV infection, with sexual behaviours that entail risk of acquiring HIV infection and, more recently and directly, with increased risk of HIV seroconversion [1-10]. Recent seroincidence studies estimate that up to 28% of contemporary HIV infections among MSM can be attributed to nitrite inhalant use [8].

Among MSM studied in San Francisco between 1985 and 1991, the prevalence of poppers use varied annually from 29.9% to 35.9% [7]. Consistent users of poppers were 2.5 times (95% CI 1.2 – 4.9) more likely to seroconvert than non-users. Among 3257 MSM surveyed in 6 US cities between 1995 and 1997, use of poppers during the previous 6 months was reported at 26.9% of study visits and in multivariate analyses, was associated with a 2.2-fold (95% CI 1.4–3.7) increased risk of HIV seroconversion [8]. More recently, we reported a similar 2.2-fold unadjusted relative hazard for HIV seroconversion associated with use of poppers in an open cohort of young MSM [9].

Detailed studies of nitrite use among MSM during the past 5 years are scarce. One random digit dial survey conducted in 2003 reported that poppers were used during the previous six months by 18.6% of HIV-seronegative MSM residing in Seattle, Washington [4]. Use was significantly more common among men reporting recent unprotected anal intercourse with a sexual partner whose HIV status was positive or unknown than among men not reporting such unprotected sex (28.9% of 45 vs. 6.2% of 195), though in multivariate analyses, this association approached but was not statistically significant (OR = 2.7, 95% CI 0.9–8.0).

The substantial contribution of nitrite inhalants to contemporary HIV infections among MSM is well established. Remarkably, in recent years mention of nitrite inhalants in HIV prevention messages has become exceedingly rare. Risk reduction counselling guidelines do not include clear and specific messages about the increased risk for acquiring HIV infection associated with nitrite use [11,12]. Indeed, it is no longer clear whether HIV-seronegative men even receive specific counselling or appreciate these risks associated with nitrite inhalants.

In the province of British Columbia, annual reports of new HIV diagnoses among MSM from 1999 and 2004 increased 78% (from 95 to 169) [13,14].

This tremendous increase in HIV infections among MSM in BC, the importance of poppers as a modifiable risk factor for HIV seroconversion, and the scarcity of recent related research prompted us to study the prevalence and correlates of contemporary nitrite inhalant use among young MSM in Vancouver.

## Methods

The Vanguard Project was a prospective, open cohort study of HIV incidence and risk behaviours among young MSM [15,16]. Briefly, HIV-seronegative self-identified gay and bisexual men between 18 and 30 years of age who lived in Vancouver were recruited at community events, in community clinics and through advertisements in local gay newspapers. At baseline and annually thereafter, participants underwent HIV serologic testing with pre- and post-test counselling and completed a confidential self-administered questionnaire. Written informed consent was obtained from participants according to a protocol approved by the University of British Columbia Research Ethics Board.

Results from our previous study showed the association of substance use and unprotected anal intercourse (UAI) depends on the type of drug-use measure (any use vs. use specifically during sexual encounters), type of sexual partner (casual vs. regular) and sexual position (insertive vs. receptive) [5]; therefore, we examined the association between high-risk sexual behaviours and nitrite inhalant use with these distinctions in mind. The present analysis was restricted to the eighth and final wave of data collection, October 2002 through May 2004. Participants were asked whether they had engaged in any protected and/or unprotected anal intercourse (receptive, insertive, or both) during the previous year with regular and casual partners, respectively. Regular partners were defined as those with whom the participant had sex with at least once a month; casual partners were those with whom the participant had sex with less than once a month. Men were asked to report numbers of regular and casual partners, by partners' HIV serostatus (positive, negative, unknown). Men reported any use of licit and illicit drugs during the previous year. In addition, they specified those drugs used shortly before or during sex with regular and casual partners, respectively, stratified by partners' HIV serostatus.

We compared men who did and did not report use of nitrite inhalants using Pearson's chi-square, Fisher's exact, and Wilcoxon rank-sum tests. Correlates of use were identified using odds ratios (OR) with exact 95% confidence intervals (CI) computed using PEPI for Windows software Version 4.18 [17].

## Results

Among 354 MSM study participants, 66.4% were white, 13.6% were Canadian-Aboriginal, 51.3% had completed college, 51.1% were employed full-time, and 7.0% were HIV-seropositive. Use of poppers during the previous year was reported by 112 men (31.6%). Compared to non-users, men who used poppers were younger [median age 30, (interquartile range, IQR 26.5, 340) vs. 32, (IQR 29, 35), p = 0.003], more likely to have stable housing (92.0% vs. 83.2% p = 0.032), and more likely to report an annual income greater than CDN$10,000 (89.2% vs. 78.8%, p = 0.028). Previous-year use of poppers was not significantly associated with race-ethnicity, having completed high school or college, full time employment, or HIV serostatus.

Compared to men who did not report nitrite inhalant use, users were more likely to also report previous-year use of other substances, including alcohol (96.4% vs. 82.4%), marijuana (73.2% vs. 53.7%), ecstasy (48.2% vs. 21.5%), crystal methamphetamine (39.5% vs. 21.1%), gamma hydroxybutrate (GHB) (28.0% vs. 7.5%) and ketamine (22.2% vs. 8.8%) (all p < 0.001), as well as cocaine (39.5% vs. 23.0%, p = 0.002). Men who did and did not report use of poppers were not significantly different with regard to previous-year use of cigarettes, crack cocaine, amphetamines (other than crystal methamphetamine), lysergic acid (LSD), or heroin. Compared to non-users, men who used nitrite inhalants were more likely to have met sexual partners in bars (53.6% vs. 34.0%), on the Internet (45.5% vs. 27.1%), and in bathhouses (37.5% vs. 17.6%) (all p < 0.001).

Among 349 men (99%) who answered the question, previous-year use of poppers during sexual encounters was reported by 80 (22.9%). In the sample overall, men who used poppers during sexual encounters were significantly more likely to report unprotected receptive anal intercourse with a casual partner [25 (31%) of 80 vs. 26 (10%) of 269, OR = 4.0, 95% CI 2.2–8.3)]. However, this association could indicate that men who use poppers during sexual encounters are more likely to have a casual partner, or are more likely to have anal intercourse with a casual partner, or are more likely to have receptive than insertive intercourse with a casual partner, or are less likely to use a condom during anal receptive encounters with casual partners. To explore each of these possibilities, we progressively restricted the sample to examine each association in turn.

Compared to non-users, men reporting nitrite inhalant use during sexual encounters had a 17-fold increased likelihood of having casual partners (Table 1) and greater numbers of them (median 8 vs. 4, p < 0.001). Among the 234 men with casual partners, users of poppers had greater numbers of them during the previous year, including partners whose HIV status was unknown (median 7 vs. 4, p = 0.0001), seronegative (median 5 vs. 2, p = 0.004) and seropositive (median 2 vs. 1, p = 0.072).

**Table 1:** Characteristics of study participants, by self-reported previous-year use of poppers during sexual intercourse with casual partners (n = 349)

	**Poppers use with casual partners**		
	**Yes (n = 80)** **No. (%)**	**No (n = 269)** **No. (%)**	**Odds Ratio ** **(95% CI)**

Has casual partner(s)	75 (96.2)	159 (59.1)	17.3 (5.4–87.5)
No casual partner(s)	3 (3.9)	110 (40.9)	
			
Any anal intercourse with casual partner	70 (93.3)	103 (67.3)	6.8 (2.5–22.8)
No anal intercourse with casual partner	5 (6.7)	50 (32.7)	
			
Receptive anal intercourse (RAI) with casual partner	58 (77.3%)	75 (50.0%)	3.4 (1.8–6.8)
No RAI with casual partner	17 (22.7%)	75 (50.0%)	
			
Unprotected RAI with casual partner	25 (43.1)	26 (34.7)	1.4 (0.7–3.1)
No unprotected RAI with casual partner	33 (56.9)	49 (65.3)	
			
Insertive anal intercourse (IAI) with casual partner	60 (80.0)	88 (57.9)	2.9 (1.5–6.0)
No IAI with casual partner	15 (20.0)	64 (42.1)	
			
Unprotected IAI with casual partner	29 (48.3)	29 (33.0)	1.9 (0.9–3.9)
No unprotected IAI with casual partner	31 (51.7)	59 (67.1)	

Furthermore, among men with casual partners, those who used poppers were almost seven times more likely than non-users to report engaging in anal intercourse with these partners (Table 1), the odds being similarly increased for insertive (OR = 2.9) as for receptive (OR = 3.4) anal intercourse (Table 1). In contrast, once analyses were restricted to those 148 and 133 men reporting insertive and receptive intercourse with casual partners, respectively, use of poppers was much less strongly and non-significantly associated with unprotected encounters of either sort (Table 1).

In summary, use of poppers during sexual encounters was strongly associated both with having greater numbers of casual partners (of every HIV serostatus) and with engaging in anal intercourse with casual partners. However, among men who reported anal intercourse with casual partners, use of poppers during these encounters was not significantly associated with non-use of condoms during these encounters.

With regular partners, associations between the use of poppers and unprotected sexual behaviours were markedly different. In the overall sample, use of poppers during these encounters was associated with neither insertive nor receptive unprotected anal intercourse [26 (32.5%) of 80 vs. 105 (39.0%) of 269, OR = 0.8, 95% CI 0.4–1.3 and 30 (37.5%) of 80 vs. 108 (40.2%) of 269, OR = 0.9, 95% CI 0.5–1.5, respectively]. Similarly, in analyses progressively restricted like those used to examine sexual behaviours with casual partners, use of poppers during encounters with regular partners was not associated with any of the behaviours measured, except for a negative association with unprotected insertive anal intercourse (Table 2).

**Table 2:** Characteristics of study participants, by self-reported previous-year use of poppers during sexual intercourse with regular partners (n = 349)

	**Poppers use with regular partners**		
	**Yes (n = 80) ** **No. (%)**	**No (n = 269) ** **No. (%)**	**Odds Ratio** ** (95% CI)**

Has regular sex partner	54 (67.5)	184 (68.4)	1.0 (0.5–1.7)
No regular sex partner	26 (32.5)	85 (31.6)	
			
Any anal intercourse with regular partner	51 (94.4)	154 (86.5)	2.6 (0.8–14.3)
No anal intercourse with regular partner	3 (5.6)	24 (13.5)	
			
Receptive anal intercourse (RAI) with regular partner	46 (85.2)	140 (79.6)	1.5 (0.6–3.9)
No RAI with regular partner	8 (14.8)	36 (20.5)	
			
Unprotected RAI with regular partner	30 (65.2)	108 (77.1)	0.6 (0.3–1.2)
No unprotected RAI with regular partner	16 (34.8)	32 (22.9)	
			
Insertive anal intercourse (IAI) with regular partner	45 (83.3)	131 (75.3)	1.6 (0.7–4.1)
No IAI with regular partner	9 (16.7)	43 (24.7)	
			
Unprotected IAI with regular partner	26 (57.8)	105 (80.2)	0.3 (0.2–0.8)
No unprotected IAI with regular partner	19 (42.2)	26 (19.9)	

## Discussion

In the present study, we investigated use of nitrite inhalants in a large, community-recruited cohort of young MSM during a period of rapidly increasing HIV incidence in British Columbia. We found that nearly one third of men reported using poppers during the previous year and that nearly one in four reported using poppers during sexual encounters. Men who used poppers during sexual encounters were much more likely than non-users to have casual partners; to have greater numbers of casual partners whose HIV serostatus was positive or unknown; and to have anal intercourse with casual partners. However, among men who engaged in receptive and insertive anal intercourse with their casual partners, the use of poppers during these encounters was not significantly associated with the encounter being unprotected. Thus, among young MSM we studied, reported use of poppers during sexual encounters was a very strong indicator of engaging in anal intercourse with high-risk casual partners but not a significant determinant of condom use per se.

The prevalence of nitrite use in our cohort is similar to the 25–35% reported in the small number of related studies of MSM published since the introduction of highly active antiretroviral therapy [4,8,10,18-20]. It is particularly noteworthy that the prevalence of nitrite use we observed in 2002–2004 appears unchanged from that reported by the same cohort in 1995–1996 (34%); by comparison, the prevalence of nitrite use in an independent cohort of MSM in Vancouver in 1985 was 43% [21].

As in previous studies, we observed a strong association between use of poppers and unprotected anal intercourse [4,10,18-20]. Unlike previous studies however, we progressively restricted analyses to determine whether use of nitrites is merely an indicator of having anal intercourse with casual sexual partners or a determinant of condom use per se; our results are more consistent with the former than the latter.

HIV-seronegative MSM in our cohort who use poppers were more likely than non-users to engage in anal intercourse with casual partners, including those whose HIV serostatus is positive or unknown. Previous studies suggest that poppers confer an increased risk for HIV seroconversion via a physiological mechanism: increased risk for infection following sexual exposure to the virus. Together, these studies suggest that the high risk for HIV seroconversion among MSM who use poppers reflects a synergism between two risks: more frequent encounters with infected partners and a higher probability of infection following each exposure.

Nitrite inhalant use by men in our study occurred during a rapid increase in HIV diagnoses among MSM in BC, underscoring the urgent need to reduce use of these substances. It is noteworthy that others estimate that up to 28% of contemporary HIV infections among MSM may be attributed to use of nitrite inhalants; this estimate reflects both the strength of the association with HIV seroconversion and the prevalence of nitrate inhalant use among MSM [8]. Most studies report a somewhat higher risk for HIV seroconversion associated with the use of methamphetamine than with the use of poppers. We wish to emphasize that the greater prevalence of poppers use indicates that in most settings, more incident HIV infections among MSM can be attributed to use of nitrite inhalants than to use of methamphetamine.

Strengths of the present study include our community-based recruitment of a large sample of young MSM; our distinction between any use of poppers and use during sexual encounters specifically, between casual and regular partners, and between insertive versus receptive anal intercourse; our assessment of perceived HIV serostatus of sexual partners; and the precision with which we relate use of nitrite inhalants and use of condoms during sexual encounters with casual partners. Our study also has several limitations that should be kept in mind. High-risk sexual and substance use behaviours are self-reported and subject to under-reporting. Ours was not a random sample of MSM in Vancouver and these results may not apply to other MSM, particularly those who are older or HIV-seropositive. We did not measure and so are unable to relate use of poppers with less commonly reported sexual behaviours (for example, fisting and group sex) or with use of condoms during specific acts of sexual intercourse. The latter limitation is common to all but a handful of studies that relate high-risk sexual behaviours and drug use [5].

## Conclusion

In summary, we report a disturbingly high prevalence of nitrite inhalant use among young MSM during a period of rapidly increasing HIV incidence in British Columbia. Our results, together with those from previous studies, suggest nitrite inhalant use by MSM is associated with a synergy among risks for HIV seroconversion. Use of poppers is associated with an increased likelihood of engaging in anal intercourse with an infected partner and in addition, a higher probability of infection following each such exposure. Rapid assessments are needed to determine whether MSM are aware of HIV-related risks associated with use of poppers. Efforts to reduce the use of nitrite inhalants during sexual encounters should be considered a high-priority HIV prevention strategy for MSM.

## Competing interests

The author(s) declare that they have no competing interests.

## Authors' contributions

TML conceived and designed the study, supervised data analyses and revised drafts of the manuscript. KM reviewed literature, produced the first draft of the manuscript and, with KC, performed data analyses and manuscript revisions. RSH reviewed a draft of the manuscript. All authors read and approved the final version of the manuscript.

## Pre-publication history

The pre-publication history for this paper can be accessed here:


